# Developments in Electrical-Property Tomography Based on the Contrast-Source Inversion Method

**DOI:** 10.3390/jimaging5020025

**Published:** 2019-02-01

**Authors:** Reijer Leijsen, Patrick Fuchs, Wyger Brink, Andrew Webb, Rob Remis

**Affiliations:** 1 Department of Radiology, C.J. Gorter Center for High Field MRI, Leiden University Medical Center, 2333ZA Leiden, The Netherlands; 2 Circuits and Systems Group, Delft University of Technology, Faculty of Electrical Engineering, Mathematics and Computes Science, 2628CD Delft, The Netherlands

**Keywords:** electromagnetic inverse scattering problems, magnetic resonance imaging, electrical-property tomography, nonlinear optimization, contrast-source inversion

## Abstract

The main objective of electrical-property tomography (EPT) is to retrieve dielectric tissue parameters from ${\hat{B}}_{1}^{+}$ data as measured by a magnetic-resonance (MR) scanner. This is a so-called hybrid inverse problem in which data are defined inside the reconstruction domain of interest. In this paper, we discuss recent and new developments in EPT based on the contrast-source inversion (CSI) method. After a short review of the basics of this method, two- and three-dimensional implementations of CSI–EPT are presented along with a very efficient variant of 2D CSI–EPT called first-order induced current EPT (foIC-EPT). Practical implementation issues that arise when applying the method to measured data are addressed as well, and the limitations of a two-dimensional approach are extensively discussed. Tissue-parameter reconstructions of an anatomically correct male head model illustrate the performance of two- and three-dimensional CSI–EPT. We show that 2D implementation only produces reliable reconstructions under very special circumstances, while accurate reconstructions can be obtained with 3D CSI–EPT.

## 1. Introduction

The conductivity and permittivity values of different tissue types are of great importance in a variety of medical applications. In magnetic-resonance (MR) safety [1] and hyperthermia-treatment planning [2], for example, conductivity tissue profiles are required to determine the specific absorption rate (SAR). Conductivity may also serve as a biomarker in oncology or in acute-stroke imaging [3]. Permittivity is important since it affects the spatial distribution of the transmitted electromagnetic field responsible for spin excitation.

Typically, tissue conductivity and permittivity values are measured ex vivo for a particular range of frequencies [4]. Other methods require elaborate hardware, such as electrical impedance tomography (EIT) [5] or microwave-imaging methods [6]. The objective of electrical-property tomography (EPT) is to retrieve these dielectric tissue values in vivo using an MR scanner and standard measurement protocols [3,7]. Specifically, with an MR scanner, the so-called ${\hat{B}}_{1}^{+}$-field, defined as ${\hat{B}}_{1}^{+}=\left({\hat{B}}_{x}+j{\hat{B}}_{y}\right)/2$, can be measured at a particular frequency of an operation called the Larmor frequency. This frequency is proportional to the magnitude of static background field $B_{0}$ via relation *f* = *γB*_0_, where *γ* $=42.577\;\mathrm{MHz}\;T^{-1}$ is the proton gyromagnetic ratio divided by 2π, leading to MR operating frequencies of 128 and 298 MHz for a 3 and 7 T scanner, respectively.

Reconstruction of dielectric tissue parameters is based on the measured ${\hat{B}}_{1}^{+}$-field, and what sets EPT apart from other more common inversion and imaging problems is that the measured ${\hat{B}}_{1}^{+}$-field has its support inside the reconstruction domain. The EPT reconstruction problem therefore belongs to the class of so-called hybrid inverse problems [8], and several EPT techniques have been proposed to reconstruct the conductivity and permittivity profiles based on these internal ${\hat{B}}_{1}^{+}$ data. Loosely speaking, these techniques can be divided into local differential-based approaches (see, e.g., References [9,10,11,12]) and global integral-based approaches (see e.g., References [13,14,15,16,17,18]). Combinations of local and global methods have been developed as well [19,20].

In this paper, we focus on a global integral-based EPT reconstruction method called contrast source inversion (CSI)–EPT, where a CSI approach [21,22,23] is taken to solve an EPT reconstruction problem. In particular, in CSI–EPT, the reconstruction problem is formulated as an optimization problem in which an objective function is iteratively minimized. This objective function consists of a term that measures the mismatch between modeled and measured data (data mismatch), and a term that measures the discrepancy in satisfying Maxwell’s equations within the reconstruction domain using a global integral field representation (consistency mismatch). Including the second consistency term in the objective function is crucial to the performance of CSI, as shown in Reference [24].

Minimization of the objective function is carried out by iteratively updating a contrast function, which describes the dielectric constitution of the body part of interest, and a so-called contrast source, which is the product of the contrast function and the electric-field strength. Updating takes place by fixing one variable and updating the other. More precisely, the contrast function is first fixed, the contrast source is updated, and subsequently the contrast source is fixed and the contrast function is updated.

The CSI–EPT method was originally introduced in Reference [14], where it was shown that CSI–EPT is able to reconstruct strongly inhomogeneous conductivity and permittivity profiles within the center slice of an object placed in the center of a body coil in a 3 T MR scanner. The method was initially implemented for E-polarized electromagnetic fields in two-dimensional (2D) configurations in which the electrical field is parallel to the bore axis (*z*-axis) and the magnetic field is purely transverse, because it is significantly less complex than full three-dimensional implementation. The use of a 2D approach was justified since it was shown that the electromagnetic field in the midplane of a birdcage coil essentially has an E-polarized field structure [25]. An efficient alternative to CSI–EPT, first-order induced-current EPT or foIC-EPT, was presented in Reference [20] as well. This method exploits the structure of the two-dimensional E-polarized field to efficiently reconstruct the tissue profiles in the midplane of the transmit coil. The foIC-EPT method is significantly faster than CSI–EPT and produces reconstructions in real time with essentially the same quality as 2D CSI–EPT.

The CSI–EPT method has recently been extended to three-dimensional (3D) configurations in Reference [26]. With this 3D implementation of CSI–EPT, volumetric conductivity and permittivity profiles are obtained, and it is no longer necessary to restrict the reconstruction domain to the midplane of a transmit coil. Moreover, 3D CSI–EPT is based on vectorial 3D Maxwell equations and no (E-polarized) field structure is assumed to be present as in the case of a 2D approach. Unfortunately, computation times dramatically increase compared with 2D CSI–EPT and foIC-EPT and, depending on the configuration, it may take 3D CSI–EPT hours or even days to converge even on dedicated high-performance computers or servers. Apart from possible preconditioning techniques that may be applied to accelerate the convergence of 3D CSI–EPT, 2D CSI–EPT or foIC-EPT may be preferable in practice, since reconstruction times are significantly shorter compared with 3D approaches.

In this paper, we thoroughly investigate this issue and compare reconstructions obtained with 2D CSI–EPT, foIC-EPT, and 3D CSI–EPT. Reconstruction artifacts in the conductivity and permittivity profiles, the modeled ${\hat{B}}_{1}^{+}$-field, and the internal electric field are carefully studied. Our analysis shows that only under very special conditions is a 2D approach justified. Even if the electromagnetic field has an E-polarized field structure in the midplane of the transmit coil, imposing a two-dimensional field structure is generally too limiting an approximation unless the body part of interest and transmit coil strictly satisfy the longitudinal invariance condition.

This paper is organized as follows. In Section 2, the 2D and 3D CSI–EPT method is briefly reviewed, and the governing integral representations are presented. A variant of 2D CSI–EPT, foIC-EPT, is also presented, and a detailed analysis of the performance of all three reconstruction methods is presented in Section 3 using a realistic head model from Virtual Family [27]. A discussion with conclusions can be found in Section 4. Finally, we note that the position vectors in 2D and 3D are denoted by ρ and x, respectively, and we use an exp(+jωt) time convention.

## 2. Theory

As mentioned above, the CSI–EPT algorithm operates on two unknowns and is based on two fundamental equations. Specifically, the unknowns in CSI–EPT are contrast function $\hat{\chi }$ andthe contrast source $\hat{w}$, and the fundamental equations are the data equation and object or state equation.

The contrast function describes the dielectric contrast of the body with respect to free space and is given by $\hat{\chi }\left(x\right)=\varepsilon_{r}\left(x\right)-1-j\sigma \left(x\right)/\omega \varepsilon_{0}$, where $\varepsilon_{r}\left(x\right)$ and σ(x) are, respectively, the unknown relative permittivity and conductivity profiles of the body, $\varepsilon_{0}$ is the permittivity of free space, and ω is the Larmor frequency of operation. The contrast function has bounded domain $D_{\mathrm{body}}$ that is occupied by the body as its support, that is, the contrast function vanishes for $x\notin D_{\mathrm{body}}$. Finally, we note that the contrast function is dimensionless, and that its real part is determined by the permittivity profile, while its imaginary part is determined by the body’s conductivity profile.

The contrast source in CSI–EPT is defined as $\hat{w}=\hat{\chi }\hat{E}$, where $\hat{E}\left(x\right)$ is the electric-field strength. Note that it is common to refer to $\hat{w}$ as a contrast source even though it is expressed in volts per meter and is actually scaled electric-field strength. The electric-field strength is obviously also unknown, since the dielectric constitution of the body is unknown. Even though this field is not of primary interest in EPT, CSI–EPT does provide electric-field reconstructions that may be used to reconstruct the local time-averaged power density that is dissipated into heat [1].

To arrive at the two fundamental equations of CSI–EPT, we set up a scattering formalism in which we make use of the linearity of Maxwell’s equations and exploit the fact that the body occupies a bounded domain $D_{\mathrm{body}}$. In particular, we first determine the electromagnetic field that is present inside an empty birdcage coil. In practice, this so-called background field is computed using electromagnetic-simulation software and we denote it by $\left\{{\hat{E}}^{b},{\hat{B}}^{b}\right\}$. We note that the assumption is made here that external currents are impressed and field-independent. Consequently, antenna loading is not directly taken into account. The total electromagnetic field in presence of the body is denoted by $\left\{\hat{E},\hat{B}\right\}$, and using the linearity of Maxwell’s equations this field can be written as

(1) $$\left\{\hat{E},\hat{B}\right\}=\left\{{\hat{E}}^{b},{\hat{B}}^{b}\right\}+\left\{{\hat{E}}^{\mathrm{sc}},{\hat{B}}^{\mathrm{sc}}\right\},$$

where $\left\{{\hat{E}}^{\mathrm{sc}},{\hat{B}}^{\mathrm{sc}}\right\}$ is the scattered electromagnetic field due to the presence of the body. For this field, we have the integral representations

(2) $${\hat{B}}^{\mathrm{sc}}\left(x\right)=\int_{x^{\prime }\in D_{\mathrm{body}}}{\underline{\hat{G}}}^{\mathrm{BJ}}\left(x,x^{\prime }\right)\cdot \hat{w}\left(x^{\prime }\right)\;dV\;\mathrm{and}\;{\hat{E}}^{\mathrm{sc}}\left(x\right)=\int_{x^{\prime }\in D_{\mathrm{body}}}{\underline{\hat{G}}}^{\mathrm{EJ}}\left(x,x^{\prime }\right)\cdot \hat{w}\left(x^{\prime }\right)\;dV,$$

where ${\underline{\hat{G}}}^{\mathrm{EJ}}$ and ${\underline{\hat{G}}}^{\mathrm{BJ}}$ are essentially the electric current to the electric field and electric current to magnetic field Green’s tensors of the background medium. Note that these are the Green’s tensors of a homogeneous background medium, and the presence of the coil is not taken into account. Explicit expressions for these tensors are given below.

Having these integral representations at our disposal, we can now present the basic CSI–EPT equations. We start with the equation that relates the measured ${\hat{B}}_{1}^{+}$-field to the contrast source. In particular, using the integral representation for the scattered magnetic field of Equation (2), we have

(3) $${\hat{B}}_{1}^{+;\mathrm{sc}}\left(x\right)=\frac{{\hat{B}}_{x}^{\mathrm{sc}}+j{\hat{B}}_{y}^{\mathrm{sc}}}{2}=\frac{1}{2}\int_{x^{\prime }\in D_{\mathrm{body}}}\sum_{k=x,y,z}\left[{\hat{G}}_{xk}^{\mathrm{BJ}}\left(x,x^{\prime }\right)+j{\hat{G}}_{yk}^{\mathrm{BJ}}\left(x,x^{\prime }\right)\right]{\hat{w}}_{k}\left(x^{\prime }\right)\;dV,$$

which can be written more compactly as

(4) $${\hat{B}}_{1}^{+;\mathrm{sc}}\left(x\right)=G_{\mathrm{data}}\left\{\hat{w}\right\}\left(x\right)\;\mathrm{for}\;x\in D_{\mathrm{body}},$$

where linear data operator $G_{\mathrm{data}}$ is implicitly defined in Equation (3). Equation (4) is known as the data equation and relates unknown contrast source $\hat{w}$ to the scattered ${\hat{B}}_{1}^{+}$-field. Note that this scattered field is known, since ${\hat{B}}_{1}^{+;\mathrm{sc}}\left(x\right)={\hat{B}}_{1}^{+}\left(x\right)-{\hat{B}}_{1}^{+;b}\left(x\right)$ and the total ${\hat{B}}_{1}^{+}$-field is known through measurements, while background field ${\hat{B}}_{1}^{+;b}\left(x\right)$ is known through simulations. The real phase is generally not known in practice, and tranceive-phase approximation is often used, which can lead to reconstruction artefacts at higher frequencies [28].

The second basic CSI–EPT equation, called the object or state equation, is obtained from the integral representation for the scattered electric field as given by the second equation of Equation (2). Using the definition of the scattered electric field ${\hat{E}}^{\mathrm{sc}}=\hat{E}-{\hat{E}}^{b}$, this integral representation can be written as

(5) $$\hat{E}\left(x\right)-\int_{x^{\prime }\in D_{\mathrm{body}}}{\underline{\hat{G}}}^{\mathrm{EJ}}\left(x,x^{\prime }\right)\cdot \hat{w}\left(x^{\prime }\right)\;dV={\hat{E}}^{b}\left(x\right)$$

and multiplying the above equation by contrast function $\hat{\chi }$, we arrive at

(6) $$\hat{w}\left(x\right)-\hat{\chi }\left(x\right)\int_{x^{\prime }\in D_{\mathrm{body}}}{\underline{\hat{G}}}^{\mathrm{EJ}}\left(x,x^{\prime }\right)\cdot \hat{w}\left(x^{\prime }\right)\;dV=\hat{\chi }\left(x\right){\hat{E}}^{b}\left(x\right)\;\mathrm{for}\;x\in D_{\mathrm{body}},$$

which can be written more compactly as

(7) $$\hat{w}\left(x\right)-\hat{\chi }\left(x\right)G_{\mathrm{body}}\left\{\hat{w}\right\}=\hat{\chi }\left(x\right){\hat{E}}^{b}\left(x\right)\;\mathrm{for}\;x\in D_{\mathrm{body}},$$

where linear operator $G_{\mathrm{body}}$ is implicitly defined in Equation (6).

To summarize, the two fundamental unknowns in CSI–EPT are contrast function $\hat{\chi }$ and contrast source $\hat{w}$, and the basic CSI–EPT equations are the data Equation (4) and the object Equation (7).

Now, suppose we have available an approximation for the contrast function and contrast source. We denote these approximants by $\tilde{\chi }$ and $\tilde{w}$, respectively, and, in order to measure how well these approximations satisfy the data and object equations, we introduce the data and object residuals as

(8) $${\hat{r}}_{d}\left(x\right)={\hat{B}}_{1}^{+;\mathrm{sc}}\left(x\right)-G_{\mathrm{data}}\left\{\tilde{w}\right\}\left(x\right)\;\mathrm{for}\;x\in D_{\mathrm{body}},$$

and

(9) $${\hat{r}}_{o}\left(x\right)=\tilde{\chi }\left(x\right){\hat{E}}^{b}\left(x\right)-\tilde{w}\left(x\right)+\tilde{\chi }\left(x\right)G_{\mathrm{body}}\left\{\tilde{w}\right\}\left(x\right)\;\mathrm{for}\;x\in D_{\mathrm{body}},$$

respectively, and measure their magnitudes using $L_{2}$-norms

(10) $$∥{\hat{r}}_{d}{∥}_{\mathrm{body}}^{2}=\int_{x\in D_{\mathrm{body}}}|{\hat{r}}_{d}{\left(x\right)|}^{2}\;dV\;\mathrm{and}\;∥{\hat{r}}_{o}{∥}_{\mathrm{body}}^{2}=\int_{x\in D_{\mathrm{body}}}{\left|{\hat{r}}_{o}\left(x\right)\right|}^{2}\;dV.$$

In CSI–EPT, these norms are used to define objective function

(11) $$F\left(\tilde{\chi },\tilde{w}\right)=\frac{∥{\hat{r}}_{d}{∥}_{\mathrm{body}}^{2}}{∥{\hat{B}}_{1}^{+;\mathrm{sc}}{∥}_{\mathrm{body}}^{2}}+\frac{∥{\hat{r}}_{o}{∥}_{\mathrm{body}}^{2}}{∥\tilde{\chi }{\hat{E}}^{b}{∥}_{\mathrm{body}}^{2}}$$

and the goal is to find a contrast function and contrast source that minimizes this objective function. We note that including the two-norm of the object residual in the objective function (second term on the right-hand side of Equation (11)) is crucial to the success of CSI, since it has been shown that a contrast-source inversion approach without this term produces unsatisfactory results in general [24].

In CSI–EPT, finding the desired contrast function is now realized by minimizing the objective function in a “fix-one-minimize-for-the-other” approach. The iterative process continues until a predefined maximum number of iterations or specified tolerance level of the objective function has been reached. Specifically, the basic CSI–EPT algorithm is as shown in Listing 1.

**Listing 1.** Contrast-source inversion–electrical-property tomography (CSI–EPT).
Given initial guesses ${\tilde{\chi }}^{\left[0\right]}$ and ${\tilde{w}}^{\left[0\right]}$ for the contrast function and contrast source, respectively,For k=1,2…
Fix the contrast to ${\tilde{\chi }}^{\left[k-1\right]}$ and update the contrast source according to the update formula
$${\tilde{w}}^{\left[k\right]}={\tilde{w}}^{\left[k-1\right]}+\alpha^{\left[k\right]}v^{\left[k\right]}.$$Compute the corresponding electric-field strength ${\hat{E}}^{\left[k\right]}$ according to (cf. Equation (5))
$${\tilde{E}}^{\left[k\right]}\left(x\right)={\hat{E}}^{b}\left(x\right)+G_{\mathrm{body}}\left\{{\tilde{w}}^{\left[k\right]}\right\}\left(x\right).$$Knowing contrast source ${\tilde{w}}^{\left[k\right]}$ and corresponding electric-field strength ${\tilde{E}}^{\left[k\right]}$, determine contrast function ${\tilde{\chi }}^{\left[k\right]}$ from constitutive relation ${\tilde{w}}^{\left[k\right]}={\tilde{\chi }}^{\left[k\right]}{\tilde{E}}^{\left[k\right]}$ by solving least-squares problem $∥\tilde{\chi }{\tilde{E}}^{\left[k\right]}-{\tilde{w}}^{\left[k\right]}{∥}_{\mathrm{body}}^{2}$ for minimum norm contrast function $\tilde{\chi }$.Stop if objective function is smaller than user-specified tolerance level, or if maximum number of iterations have been reached.End.


Polak–Ribière update directions are usually taken for update direction $v^{\left[k\right]}$ in Step 1 of the algorithm, but Fletcher–Reeves or Hesteness–Stiefel update directions may be used as well. To determine these update directions, the gradient of $F({\tilde{\chi }}^{\left[k-1\right]},\tilde{w})$ with respect to $\tilde{w}$ at $\tilde{w}={\tilde{w}}^{\left[k-1\right]}$ is required. Explicit expressions for this gradient and corresponding step length $\alpha^{\left[k\right]}$ can be found in Reference [23], for example.

Note also that, with Equation (5), the object residual can be written as ${\hat{r}}_{o}=\tilde{\chi }\tilde{E}-\tilde{w}$ and, in Steps 2 and 3, we find minimum-norm contrast function $\tilde{\chi }$ for which $∥{\hat{r}}_{o}{∥}_{\mathrm{body}}^{2}$ is minimized. This contrast function is generally sensitive to small perturbations in $\tilde{w}$ at locations where the magnitude of the electric field strength is “small.” To suppress this effect, we can alternatively update the contrast function at every iteration according to update formula

(12) $${\tilde{\chi }}^{\left[k\right]}={\tilde{\chi }}^{\left[k-1\right]}+\beta^{\left[k\right]}u^{\left[k\right]},$$

with $u^{\left[k\right]}$ the Polak–Ribière update direction for the contrast function, and $\beta^{\left[k\right]}$ its corresponding update coefficient. Such an approach usually has a regularizing effect and typically leads to smoother reconstructions.

### 2.1. Object and Data Operators in Three-Dimensional CSI–EPT

In three dimensions and with air as a background medium, the integral representations for the scattered fields, as given by Equation (2), take on form

(13) $${\hat{B}}^{\mathrm{sc}}\left(x\right)=j\frac{\omega }{c_{0}^{2}}\nabla \times {\hat{A}}^{\mathrm{sc}}\left(x\right)\;\mathrm{and}\;{\hat{E}}^{\mathrm{sc}}\left(x\right)=\left(k_{0}^{2}+\nabla \nabla \cdot \right){\hat{A}}^{\mathrm{sc}}\left(x\right),$$

where $c_{0}$ is the electromagnetic-wave speed in vacuum, $k_{0}=\omega /c_{0}$ the wave number in vacuum, and ${\hat{A}}^{\mathrm{sc}}$ is the vector potential given by

(14) $${\hat{A}}^{\mathrm{sc}}\left(x\right)=\int_{x^{\prime }\in D_{\mathrm{body}}}\hat{G}\left(x-x^{\prime }\right)\hat{w}\left(x^{\prime }\right)\;dV,$$

with $\hat{G}$ the three-dimensional Green’s function of the vacuum background domain given by

(15) $$\hat{G}\left(x\right)=\frac{\exp(-jk_{0}|x|)}{4\pi |x|}.$$

Note that the nabla operators act on position vector x and not on integration variable $x^{\prime }$. Three-dimensional object operator $G_{\mathrm{body}}$ can easily be identified from the second part in Equation (13). For data operator $G_{\mathrm{data}}$, however, we have to substitute the *x* and *y* components of the scattered magnetic flux density in Equation (3) to obtain

(16) $${\hat{B}}_{1}^{+;\mathrm{sc}}=\frac{\omega }{c_{0}^{2}}\left(\partial^{+}{\hat{A}}_{z}^{\mathrm{sc}}-\partial_{z}{\hat{A}}^{+;\mathrm{sc}}\right),$$

where $\partial^{+}=\frac{1}{2}\left(\partial_{x}+j\partial_{y}\right)$ and ${\hat{A}}^{+;\mathrm{sc}}=\frac{1}{2}\left({\hat{A}}_{x}^{\mathrm{sc}}+j{\hat{A}}_{y}^{+;\mathrm{sc}}\right)$. From the above expression for the scattered ${\hat{B}}_{1}^{+}$-field, the 3D data operator $G_{\mathrm{data}}$ can be identified. Note the particular structure of this operator: scattered ${\hat{B}}_{1}^{+}$-field originates from a difference between the transverse variations of the longitudinal vector potential ($\partial^{+}{\hat{A}}_{z}^{\mathrm{sc}}$) and the longitudinal variations of the transverse vector potential ($\partial_{z}{\hat{A}}^{+;\mathrm{sc}}$).

### 2.2. Object and Data Operators in Two-Dimensional CSI–EPT

In various papers (see Reference [25], for example) it has been reported that the radio-frequency (RF) field in the midplane of a birdcage coil is essentially E-polarized, meaning that the electric-field strength only has a longitudinal component ($\hat{E}={\hat{E}}_{z}i_{z}$), while magnetic -flux density only has *x* and *y* components ($\hat{B}={\hat{B}}_{x}i_{x}+{\hat{B}}_{y}i_{y}$). Additionally, in a two-dimensional configuration that is invariant in the *z* direction, external electric current densities with longitudinal components only generate E-polarized fields. Identifying the currents in the rungs of the birdcage coil with these *z*-directed external current sources and denoting the slice through the object that coincides with the midplane of the birdcage coil by $S_{\mathrm{body}}$, it makes sense to assume that within this midplane the RF field is essentially two-dimensional and E-polarized with integral representations for the scattered fields given by

(17) $${\hat{B}}^{\mathrm{sc}}\left(\rho \right)=j\frac{\omega }{c_{0}^{2}}\nabla_{T}\times {\hat{A}}^{\mathrm{sc}}\left(\rho \right),\;\mathrm{and}\;{\hat{E}}^{\mathrm{sc}}\left(\rho \right)=k_{0}^{2}{\hat{A}}^{\mathrm{sc}}\left(\rho \right),$$

where ρ is the position vector in the midplane of the birdcage coil, $\nabla_{T}=i_{x}\partial_{x}+i_{y}\partial_{y}$ is the transverse nabla operator, and

(18) $${\hat{A}}^{\mathrm{sc}}\left(\rho \right)=\int_{\rho^{\prime }\in S_{\mathrm{body}}}\hat{G}\left(\rho -\rho^{\prime }\right)\hat{w}\left(\rho^{\prime }\right)\;dS$$

is the vector potential in two dimensions (and is thus expressed as a two-dimensional integral as opposed to the three-dimensional integral in the 3D case) with

(19) $$\hat{G}\left(\rho \right)=-\frac{j}{4}H_{0}^{\left(2\right)}(k_{0}\left|\rho |\right)$$

the Green’s function of the two-dimensional homogeneous background medium (air) and $H_{0}^{\left(2\right)}$ is the Hankel function of the second kind and order zero. In this two-dimensional case, object operator $G_{\mathrm{body}}$ can easily be identified from the second part of Equation (17) and does not contain a gradient-divergence operator as in the three-dimensional case. For 2D data operator $G_{\mathrm{data}}$, we have to substitute the *x* and *y* components of the magnetic-flux density as given by the first part of Equation (17) in the definition of the ${\hat{B}}_{1}^{+}$-field to obtain

(20) $${\hat{B}}_{1}^{+;\mathrm{sc}}=\frac{\omega }{c_{0}^{2}}\partial^{+}{\hat{A}}_{z}^{\mathrm{sc}}.$$

From this expression, 2D data operator $G_{\mathrm{data}}$ can now easily be identified. Comparing the two-dimensional field representation of Equation (20) with its three-dimensional counterpart of Equation (16), we observe that longitudinal spatial variations are absent in the two-dimensional case. Moreover, the vector potentials in both expressions are different, since this quantity is computed using Equation (18) in the two-dimensional case, while the three-dimensional vector potential is given by Equation (14). Differences between two- and three-dimensional CSI–EPT reconstructions are discussed further in Section 3.

### 2.3. Simplified Two-Dimensional CSI–EPT—foIC-EPT

In two dimensions, the CSI–EPT algorithm can be simplified by exploiting the particular structure of E-polarized RF fields. To make this simplification explicit, we first introduce differentiation operator $\partial^{-}=\frac{1}{2}\left(\partial_{x}-j\partial_{y}\right)$ and note that operators $\partial^{-}$ and $\partial^{+}$ essentially factor two-dimensional Laplacian $\Delta =\partial_{x}^{2}+\partial_{y}^{2}$ as

(21) $$\Delta =4\partial^{-}\partial^{+}=4\partial^{+}\partial^{-}.$$

Now, as a first step, we substitute the second part of Equation (17) in Equation (20) to obtain

(22) $${\hat{B}}_{1}^{+;\mathrm{sc}}\left(\rho \right)=\frac{1}{\omega }\partial^{+}{\hat{E}}_{z}^{\mathrm{sc}}\left(\rho \right).$$

Subsequently, we use the definition of the scattered fields to write the above expression as

(23) $${\hat{B}}_{1}^{+}\left(\rho \right)={\hat{B}}_{1}^{+;b}\left(\rho \right)+\frac{1}{\omega }\partial^{+}{\hat{E}}_{z}\left(\rho \right)-\frac{1}{\omega }\partial^{+}{\hat{E}}_{z}^{b}\left(\rho \right)$$

and, since ${\hat{B}}_{1}^{+;b}\left(\rho \right)=\frac{1}{\omega }\partial^{+}{\hat{E}}_{z}^{b}\left(\rho \right)$, this simplifies to

(24) $${\hat{B}}_{1}^{+}\left(\rho \right)=\frac{1}{\omega }\partial^{+}{\hat{E}}_{z}\left(\rho \right).$$

If we now act with the $\partial^{-}$ operator on this equation, we obtain

(25) $$\partial^{-}{\hat{B}}_{1}^{+}=\frac{1}{4\omega }\Delta {\hat{E}}_{z}$$

and since ${\hat{E}}_{z}$ satisfies $\Delta {\hat{E}}_{z}-j\omega \mu_{0}{\hat{J}}_{z}^{\mathrm{ind}}=0$ with

(26) $${\hat{J}}_{z}^{\mathrm{ind}}=\left(\sigma +j\omega \varepsilon \right){\hat{E}}_{z},$$

we arrive at

(27) $${\hat{J}}_{z}^{\mathrm{ind}}=\frac{4}{j\mu_{0}}\partial^{-}{\hat{B}}_{1}^{+}.$$

This last equation shows that, in two dimensions, induced current density is obtained (accounting for multiplication by $4/j\mu_{0}$) by acting with the $\partial^{-}$ operator on the total $B_{1}^{+}$-field. The simplified CSI–EPT method is therefore called a first-order-induced current EPT method, since a first-order differentiation of the $B_{1}^{+}$-field essentially immediately results in an image of the induced current density.

As shown in Reference [20], after the induced current density is obtained, the corresponding electric-field strength can be computed by solving a specific integral equation defined on $S_{\mathrm{body}}$. With the electric-field strength now known, the conductivity and permittivity profiles within the slice can be obtained from Equation (26). The overall first-order induced current density EPT algorithm can be summarized as presented in Listing 2.

**Listing 2.** First-Order Induced Current EPT Algorithm (foIC-EPT).
Given the measured ${\hat{B}}_{1}^{+}$-field in the midplane of the birdcage coil:
Determine the induced current density using Equation (27).Determine the corresponding electric-field strength by solving a specific integral equation (Equation (12) in Reference [20]).Knowing the induced current density and the electric-field strength, determine conductivity and permittivity profiles using Equation (26).


Further details about this algorithm can be found in Reference [20]. Finally, we note that the above algorithm is a direct noniterative EPT method and, as opposed to CSI–EPT, requires the solution of a system of equations (Step 2) to arrive at the reconstructed conductivity and permittivity profiles. Fortunately, as demonstrated in Reference [20], this system of equations can efficiently be solved using iterative solvers such as the generalized minimal residual (GMRES) method [29] and, typically, only a small number of iterations is required to reach a prescribed error.

## 3. Methods and Results

**Figure 1:**
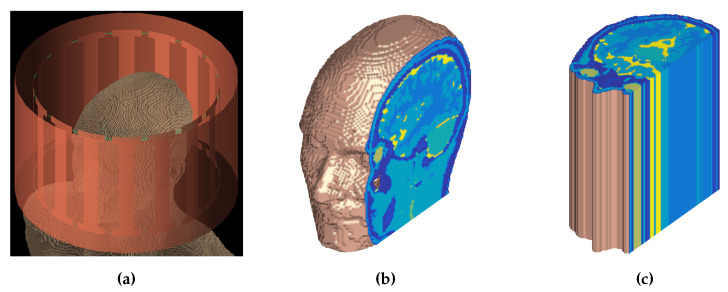
Birdcage coil and head models. (**a**) High-pass birdcage coil with the head model placed inside, (**b**) Duke head model from Virtual Family [27], and (**c**) longitudinally uniform head model obtained by repeating the center slice in the longitudinal direction.

To illustrate the performance of foIC-EPT and two- and three-dimensional CSI–EPT, we reconstructed the conductivity and permittivity profiles of the head of anatomical human-body model Duke from Virtual Family [27] (see Figure 1a,b), from noisefree ${\hat{B}}_{1}^{+}$-data. The head model consists of 124×100×109 isotropic voxels with side lengths of 2 mm. The model was placed inside an ideal high-pass birdcage coil (see Figure 1a) consisting of 16 rungs, each having a width of 25 mm. The coil has a radius of 150 mm, is 195 mm long, and is driven in quadrature at 128 MHz, which corresponds to the operating frequency of a 3 T MRI system. The shield surrounding the coil has a radius of 180 mm and length of 200 mm. Commercial EM simulation software (XFdtd, v.7.5, Remcom State College, PA, USA) was used to obtain the background field $\left\{{\hat{E}}^{b},{\hat{B}}^{b}\right\}$ as generated by the high-pass birdcage coil. Finally, to investigate the difference between two- and three-dimensional conductivity and permittivity reconstructions, we also considered a longitudinally uniform “head model” in which the center slice was simply repeated in the longitudinal direction, thereby creating a model with no variations in the longitudinal *z* direction within the head (see Figure 1c).

### 3.1. Two-Dimensional CSI–EPT and foIC-EPT

**Figure 2:**
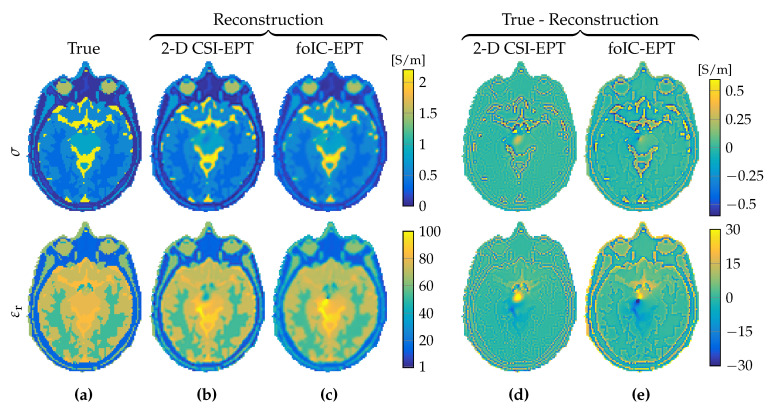
Reconstruction results from 2D reconstruction methods. (**a**) True model, (**b**) reconstruction obtained after 5000 iterations of 2D CSI–EPT, and (**c**) reconstruction from foIC-EPT. Respective errors are shown in (**d**,**e**). Top row shows conductivity and bottom row shows relative permittivity.

The CSI–EPT method was originally implemented for two-dimensional configurations in Reference [14] to study its potential as an EPT reconstruction method and to test if the method can handle strongly inhomogeneous tissue profiles. Let us therefore start with a purely two-dimensional reconstruction problem in which we attempt to reconstruct conductivity and permittivity profiles within the center slice of the head model shown in Figure 2a. In this two-dimensional setting, we took the background field in the midplane of the realistic birdcage coil shown in Figure 1a as the 2D background field. The reconstructed conductivity and permittivity profiles obtained after 5000 iterations of the two-dimensional CSI–EPT method are shown in Figure 2b. It took the algorithm approximately 86 s on an Intel i7-6700 CPU (Intel, Santa Clara, CA, USA) operating on Windows 7 with Matlab 2016a (Mathworks, Natick, MA, USA) to arrive at these reconstructions, and we terminated the algorithm after 5000 iterations since the objective function had already dropped below a $1.53\times 10^{-5}$ tolerance level at that point and essentially no significant improvements were obtained. In addition, the foIC-EPT reconstruction profiles of conductivity and permittivity are shown in Figure 2c, and the errors of CSI–EPT and foIC-EPT conductivity and permittivity reconstructions are shown in Figure 2d,e, respectively. We observed that the quality of the foIC-EPT reconstructions was similar to CSI–EPT even though it took foIC-EPT only a fraction of a second to produce these reconstructions (see Table 1 for details).

### 3.2. Three-Dimensional CSI–EPT

**Figure 3:**
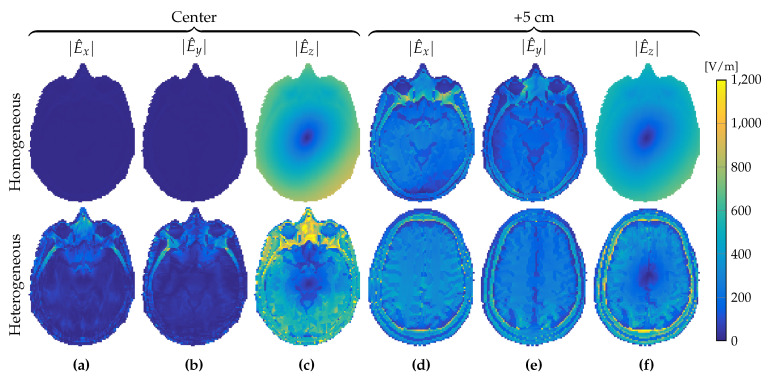
Magnitude of electric-field-strength components. *x*, *y*, and *z* components at (**a**–**c**) the transversal midplane, and (**d**–**f**) the slice 5 cm higher, respectively. Top and bottom row show fields in the case of a longitudinal homogeneous and heterogeneous object, respectively.

**Figure 4:**
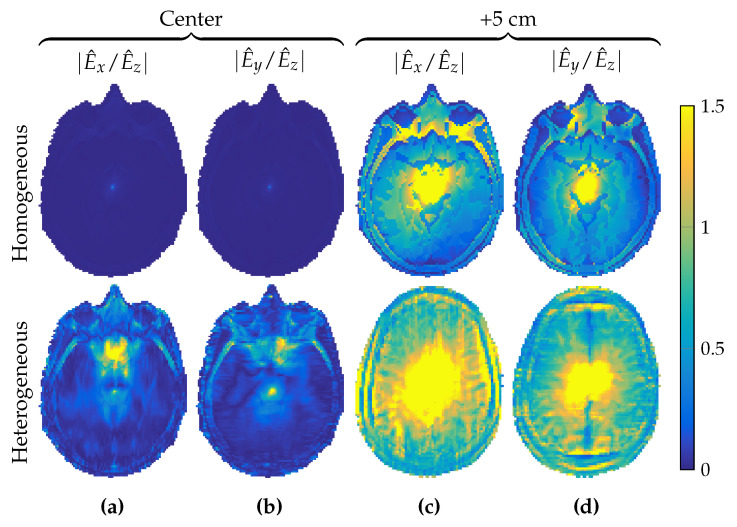
Ratios of *x* and *y* components of the electric-field strength relative to its *z* component. Relative field components at (**a**,**b**) the center slice and (**c**,**d**) the slice 5 cm higher. Top row is for the longitudinally uniform object, bottom row for the object with longitudinal variations.

In a two-dimensional approach, the RF field is E-polarized with electric-field strength that is longitudinal ($\hat{E}={\hat{E}}_{z}i_{z}$) and magnetic-flux density that is transverse ($\hat{B}={\hat{B}}_{x}i_{x}+{\hat{B}}_{y}i_{y}$). Such an approach was shown to be reasonable for a homogeneous cylindrical phantom in a central region of a body coil consisting of elementary center-fed dipole antennas in Reference [25]. Indeed, when the longitudinally uniform head model of Figure 1c was placed within our birdcage coil we also observed that the *x* and *y* components of the electric-field strength in the central transverse slice were small compared to its *z* component, as illustrated in the top rows of Figure 3a–c and Figure 4a,b.

However, as we move away from the center slice in the longitudinally uniform head model of Figure 1c, the magnitude of the *x* and *y* components of the electric-field strength starts to increase, as illustrated in the top rows of Figure 3d–f and Figure 4c,d, where the magnitude of the electric-field-strength components is shown in a slice located 5 cm above the central slice. We observed that even though the transverse components of the electric-field strength were negligible within the center slice, they could no longer be neglected when 5 cm away from it.

Furthermore, for the realistic heterogeneous head model of Figure 1b, a two-dimensional E-polarized field assumption completely failed, as shown in the bottom rows of Figure 3a–f and Figure 4a–d. In the slice 5 cm above the central slice, and even within the central slice itself, the *x* and *y* components of the electric-field strength could no longer be neglected and had to be taken into account in the full Maxwell equations to properly describe RF field behavior within the head model.

**Figure 5:**
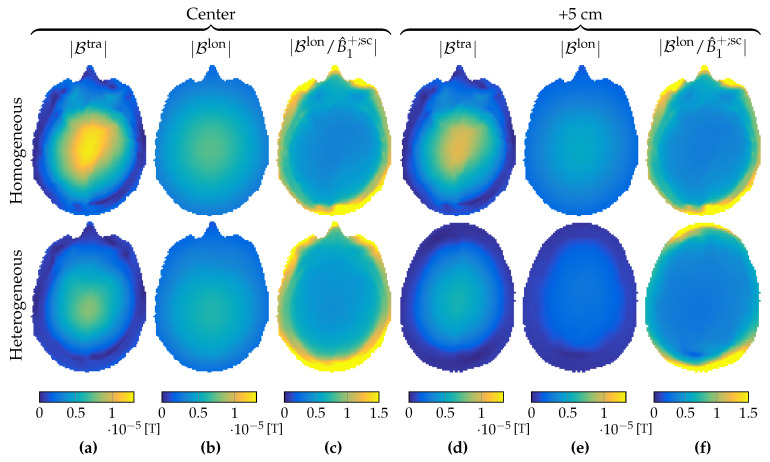
Magnitude of scattered ${\hat{B}}_{1}^{+}$ terms. (**a**) Transverse variation and (**b**) longitudinal variation term of the scattered ${\hat{B}}_{1}^{+}$, and (**c**) contributions of $B^{\mathrm{lon}}$ with regard to ${\hat{B}}_{1}^{+;\mathrm{sc}}$ at the center slice. (**d**–**f**) Same, respectively, at a slice 5 cm higher in the head domain. Top row is in the case of a longitudinally uniform object, the bottom row for the head model with longitudinal variations. (**b**) and (**e**) were neglected in the 2D approach.

To study the effects of longitudinal spatial variations of the tissue parameters on the ${\hat{B}}_{1}^{+}$-field, we considered Equation (16) again and write it in form

(28) $${\hat{B}}_{1}^{+;\mathrm{sc}}=B^{\mathrm{tra}}+B^{\mathrm{lon}},$$

where $B^{\mathrm{tra}}=\frac{\omega }{c_{0}^{2}}\partial^{+}{\hat{A}}_{z}^{\mathrm{sc}}$ and $B^{\mathrm{lon}}=-\frac{\omega }{c_{0}^{2}}\partial_{z}{\hat{A}}^{+;\mathrm{sc}}$. Longitudinal variation term $B^{\mathrm{lon}}$ is absent in a 2D approach (see Equation (20)), since, in a 2D setting, the configuration is assumed to be invariant in the longitudinal *z*-direction ($\partial_{z}=0$). Figure 5, however, shows that, for both the longitudinally homogeneous and realistic heterogeneous head model, the longitudinal variation term is significant and cannot be ignored. Especially near the periphery of both head models, $B^{\mathrm{lon}}$ contributes to the scattered ${\hat{B}}_{1}^{+}$-field. More specifically, within a 1 cm outer boundary layer located in the center slice, the mean of fraction $|B^{\mathrm{lon}}/{\hat{B}}_{1}^{+;\mathrm{sc}}|$ is 1.18 and 1.25 for the homogeneous and inhomogeneous head model, respectively; in the inner region, these means are 0.51 and 0.60, and similar averages were obtained for the slice located 5 cm above the center slice. From these observations, it is clear that longitudinal variations of transverse vector potential ${\hat{A}}^{+;\mathrm{sc}}$ contribute to the scattered ${\hat{B}}_{1}^{+}$-field and cannot be ignored.

Up to this point, we compared 3D RF field structures with their 2D counterparts for a longitudinally uniform and a realistic heterogeneous head model. In a two-dimensional configuration, however, sources are invariant in the longitudinal direction as well, and we expect that, due to the finite extent of the birdcage coil, additional deviations in the ${\hat{B}}_{1}^{+}$ fields will be observed.

**Figure 6:**
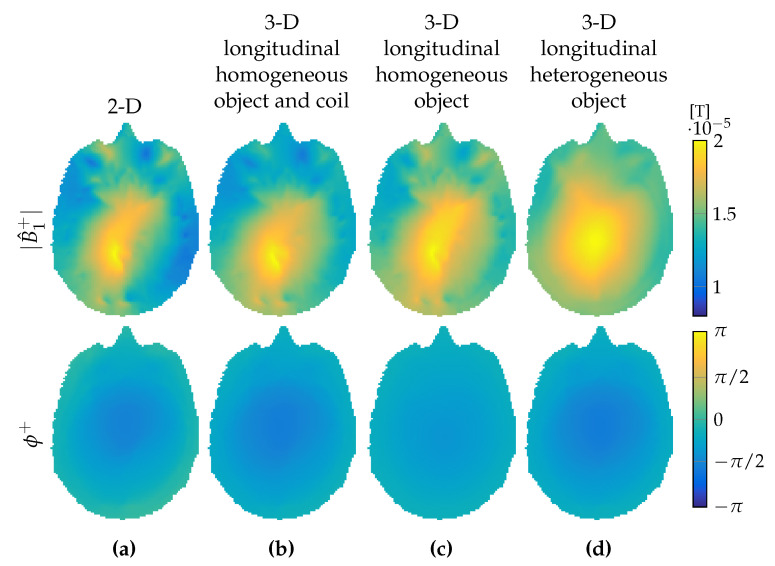
${\hat{B}}_{1}^{+}$ field comparison. (**a**) Total ${\hat{B}}_{1}^{+}$ field assumed in the 2D setting, (**b**) total ${\hat{B}}_{1}^{+}$ field obtained in a 3D setting with longitudinal homogeneity of the object and coil, (**c**) of longitudinal homogeneity of only the object, and (**d**) with longitudinal variations also of the object. Top row shows the ${\hat{B}}_{1}^{+}$ magnitude, bottom row the ${\hat{B}}_{1}^{+}$ phase.

To further investigate this issue, we first determine the two-dimensional ${\hat{B}}_{1}^{+}$-field in the central slice, as described in Section 3.1. The magnitude and phase of this field are shown in the top and bottom row of Figure 6a, respectively. Subsequently, we consider RF excitation by the 3D birdcage coil, but assume that the birdcage coil, including its currents, does not vary in the longitudinal direction. For the longitudinally uniform head model, a ${\hat{B}}_{1}^{+}$-field as shown in Figure 6b is then obtained and we observe that this field strongly resembles the 2D ${\hat{B}}_{1}^{+}$-field pattern of Figure 6a. Replacing the longitudinal invariant currents in the rungs by the exact current, but keeping the homogeneous head model, we obtain the ${\hat{B}}_{1}^{+}$-field pattern shown in Figure 6c. Agreement with the 2D field ${\hat{B}}_{1}^{+}$-field pattern clearly deteriorates, and this correspondence becomes even worse for the realistic longitudinal heterogeneous head model as shown in Figure 6d. Since the ${\hat{B}}_{1}^{+}$-field is used as an input for the CSI–EPT method, accurate correspondence is obviously necessary for proper reconstruction. The 2D CSI–EPT algorithm expects a 2D ${\hat{B}}_{1}^{+}$-field, as shown in Figure 6a for the center head slice, but in 3D the ${\hat{B}}_{1}^{+}$-field from Figure 6d is present. Providing this 3D field as an input to a 2D CSI–EPT algorithm would lead to inaccurate reconstructions in general.

**Figure 7:**
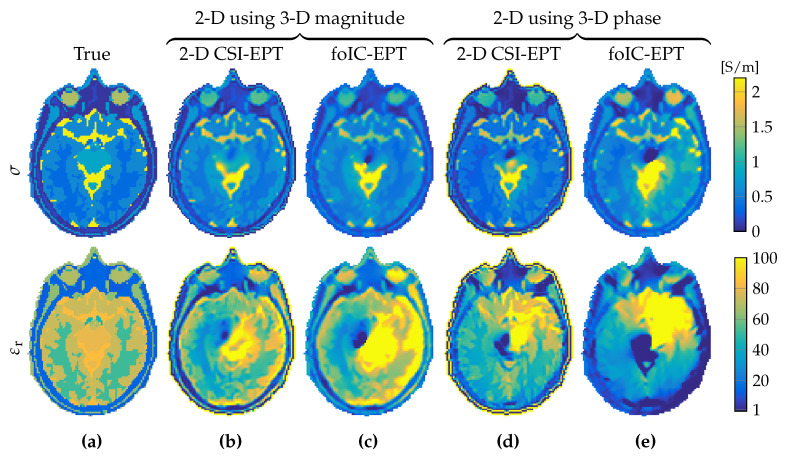
Reconstruction results from 2D reconstruction methods using parts of 3D ${\hat{B}}_{1}^{+}$ data. (**a**) True model, (**b**,**c**) reconstruction results assuming 2D phase with 3D magnitude, and (**d**,**e**) reconstruction results assuming 2D magnitude with 3D phase of the ${\hat{B}}_{1}^{+}$-field in the central transverse slice from simulations with the longitudinal invariant head model. Top row shows conductivity and the bottom row shows relative permittivity.

To illustrate how these differences in actual fields (3D) and expected fields (2D) translate to reconstruction errors, both two-dimensional algorithms were applied to quasi-three-dimensional data using either 3D amplitudes or phases. Note that, in order to match 2D and 3D data, the maximum absolute value of the ${\hat{B}}_{1}^{+}$-field of both datasets was taken to be equal. The results are depicted in Figure 7b–e, from which it can be observed that permittivity is particularly sensitive to 2D violations. This reconstruction difference between conductivity and permittivity is due to the fact that conduction currents ($\sigma \hat{E}$) influence the ${\hat{B}}_{1}^{+}$-field to a much larger extent than displacement currents ($j\omega \varepsilon \hat{E}$) at 3 T.

**Figure 8:**
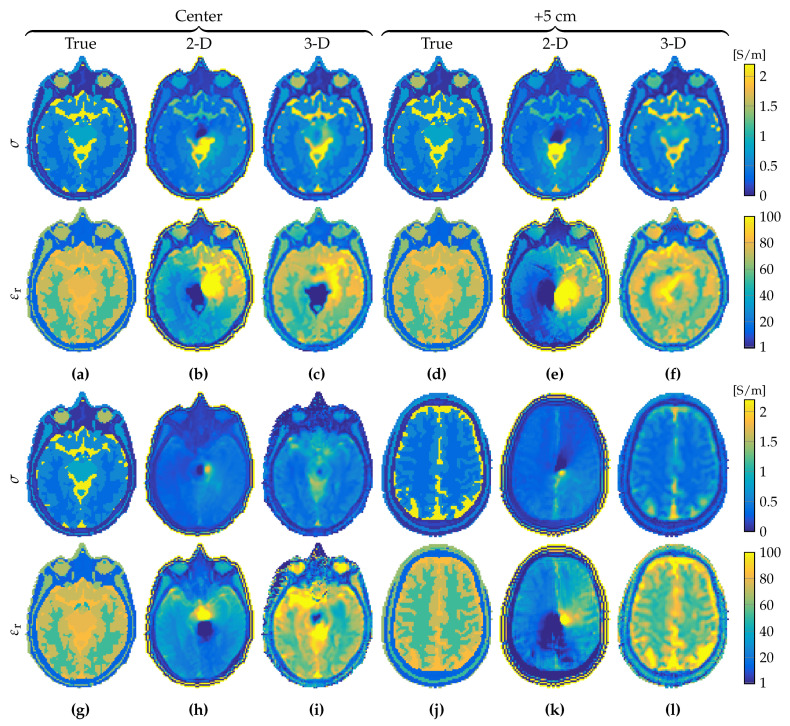
Reconstruction comparison of 2D and 3D CSI–EPT on 3D ${\hat{B}}_{1}^{+}$ fields after 5000 and 50,000 iterations, respectively. (**a**–**c**) true object, reconstruction with 2D CSI–EPT, and reconstruction with 3D CSI–EPT for a homogeneous object and for the center slice. (**d**–**f**) Same as above, but for a slice 5 cm higher. (**g**–**l**) same as (**a**–**f**), but in the case of a longitudinal inhomogeneous object. Top row depicts the conductivity, bottom row shows permittivity.

The reconstructions of the conductivity and relative permittivity profiles for the full 3D case without any further assumptions, using 3D magnitude as well 3D phase ${\hat{B}}_{1}^{+}$ data are shown for the longitudinally uniform model in Figure 8a–f and for the realistic heterogeneous head model in Figure 8g–l. Reconstructions are shown for the central slice profiles as well as for the profiles located within the slice positioned 5 cm above the central slice. For comparison, 2D CSI–EPT reconstructions based on 3D ${\hat{B}}_{1}^{+}$ data are also presented. Relative residual error (norm of the difference between the exact and reconstructed profile normalized by the norm of the exact profile, where the norm is taken over the center slice) of Figure 8h is 0.7339 and 0.8263 for the conductivity and permittivity, respectively, while the relative residual error of the conductivity and permittivity of Figure 8i is 0.3358 and 0.1587, respectively. Clearly, 2D CSI–EPT is unable to accurately reconstruct conductivity and permittivity profiles. The 2D and 3D permittivity reconstructions are also less accurate than conductivity reconstructions, indicating that ${\hat{B}}_{1}^{+}$-field data acquired at 3 T are less sensitive to permittivity variations.

**Figure 9:**
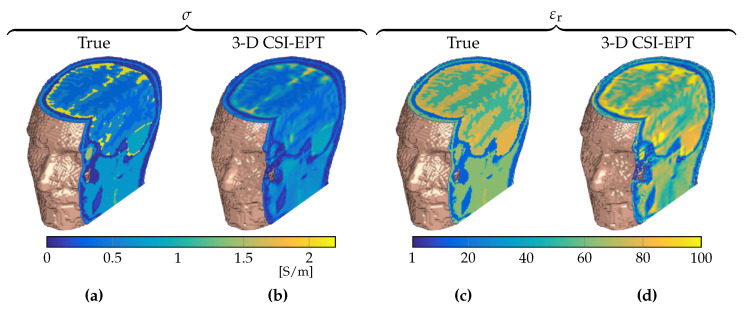
Three-dimensional visualization of a section of the 3D CSI–EPT reconstruction of the heterogeneous Duke head model (Figure 1b) after 50,000 iterations. (**a**,**b**) True and reconstructed conductivity, and (**c**,**d**) true and reconstructed relative permittivity. Top slice that is visible is the slice 5 cm above the transverse midplane.

Finally, to emphasize that 3D CSI–EPT is a fully three-dimensional volumetric reconstruction method, we present a full 3D CSI–EPT reconstruction of the realistic head model obtained after 50,000 iterations based on 3D ${\hat{B}}_{1}^{+}$ data in Figure 9. This number of iterations was chosen due to time constraints, since it takes approximately 110 h on an Intel i7-6700 CPU operating on Windows 7 with Matlab 2016a.

## 4. Discussion

We investigated the performance of two- and three-dimensional CSI–EPT in reconstructing dielectric tissue profiles based on ${\hat{B}}_{1}^{+}$ data collected inside the reconstruction slice or domain of interest. Since these data has their support inside the reconstruction domain, EPT belongs to the class of so-called hybrid inverse problems [8]. In CSI–EPT, reconstructing tissue parameters is posed as an optimization problem in which an internal objective function, that is, an objective function that measures both field and model discrepancies within the domain of interest, is minimized in an iterative manner. Field discrepancies are measured by considering the $L_{2}$-norm of the difference between modeled and measured data, while model discrepancies are measured by an $L_{2}$-norm that tells us how well a conductivity and permittivity tissue profile, and the corresponding contrast source, satisfy Maxwell’s equations. Including model discrepancies in the objective function is crucial to the performance of CSI–EPT, since it has been shown that, without this term, unsatisfactory reconstruction results may be obtained [24]. In addition to the tissue profiles, CSI–EPT reconstructs electric-field strength as well, and may therefore also be used to predict the SAR that is induced inside the body or a body part of interest [30], which is important for MR safety and hyperthermia-treatment planning, for example. Finally, we also showed that, in two dimensions, an alternative noniterative and integral-based reconstruction algorithm called foIC-EPT may be employed. This method is significantly faster than 2D and 3D CSI–EPT, and reconstructs tissue profiles and corresponding electric-field strength essentially in real time on a present-day standard laptop or PC (Intel i5-i7 or similar). However, foIC-EPT is restricted to two-dimensional configurations since it exploits two-dimensional E-polarized field structures. CSI–EPT, on the other hand, does not exploit any particular field structure and can be extended to the vectorial three-dimensional case, turning CSI–EPT into a volumetric EPT reconstruction method.

We carried out several comparisons between reconstructions obtained with 2D CSI–EPT, foIC-EPT, and 3D CSI–EPT. Our simulations show that care needs to be exercised when a 2D reconstruction approach is followed or reconstruction artefacts are obtained in the reconstructed dielectric tissue profiles. Specifically, we showed that, by using 2D methods, erroneous reconstructions could be obtained, since the longitudinal variations of the transverse vector potential are completely ignored in the data model for the ${\hat{B}}_{1}^{+}$ field. Moreover, vector potential itself is computed differently in 2D and 3D since longitudinal invariance is assumed in the 2D case. In fact, the transverse electric field and the longitudinal magnetic field vanish in 2D as a consequence of the (assumed) invariance of the object and external sources along the longitudinal direction. In 3D, however, all components of the electromagnetic field are present and their contributions to the measured data and object equations have to be taken into account. Of course, in some situations, an E-polarized field structure may be present in the midplane of a birdcage coil, but the scattered ${\hat{B}}_{1}^{+}$-field is also influenced by longitudinal variations of transverse vector potential $\partial_{z}{\hat{A}}_{z}$. These equations can only be simplified to 2D if we can guarantee that longitudinal invariance or the smoothness of certain field components can be imposed before any reconstruction algorithm is applied to the measured data. Therefore, cylindrical body parts, such as legs or arms, could be reliably reconstructed via 2D CSI–EPT, but this at least requires further validation through simulations and measurements using cylindrical phantom models with known dielectric characteristics.

No assumptions on the fields are imposed in 3D CSI–EPT and reconstruction errors due to such assumptions are therefore avoided. Moreover, 3D CSI–EPT is a volumetric reconstruction method, and is not restricted to a specific plane within the configuration. Reliable reconstructions can be obtained within any desired domain of interest provided that ${\hat{B}}_{1}^{+}$ data are available within this domain. Unfortunately, computation times significantly increase when applying 3D CSI–EPT. Depending on the number of unknowns in the EPT reconstruction problem, 3D CSI–EPT may take many iterations to converge to the desired error tolerance, with total computation times of hours or days, even on dedicated computers or servers. In future research, we focus on accelerating the convergence rate of 3D CSI–EPT by including preconditioning techniques in CSI–EPT (as described in Reference [23], for example) that exploit all a priori knowledge we have about the object or body part that needs to be reconstructed. This knowledge can also be used to construct an accurate initial guess, thereby possibly further accelerating CSI–EPT.

In our experiments, we used simulated ${\hat{B}}_{1}^{+}$-field data to test the performance of 2D and 3D CSI–EPT on strongly inhomogeneous structures, and to study the differences between two- and three-dimensional CSI–EPT approaches. In real-world measurements, the data obviously differ from simulated data, and CSI–EPT should be adapted so that it can handle measured ${\hat{B}}_{1}^{+}$ data. In this respect, we identified three practical issues that need to be addressed, which are part of our current CSI–EPT research.

First, in practice, the ${\hat{B}}_{1}^{+}$-field is obtained in polar form through separate amplitude and phase measurements. In both cases, the collected data are contaminated with noise. Therefore, filtering or regularization techniques that suppress the effects of noise should be incorporated in CSI–EPT. Initial studies show that filtering of the data allows us to handle measured data in foIC-EPT [20] and, as demonstrated in Reference [14], total-variation (TV) regularization may suppress noise effects in CSI–EPT. However, due to the many possible choices for the regularization parameter in this method, it is presently not clear for which parameter or parameter range the TV CSI–EPT scheme is most effective.

Second, the phase that is measured in practice is not the phase of the ${\hat{B}}_{1}^{+}$-field, but the so-called transceive-phase from which the ${\hat{B}}_{1}^{+}$-phase can be extracted. To this end, the tranceive-phase approximation is often applied, but the validity of this approximation is not fully understood, and may lead to reconstruction errors in conductivity and permittivity profiles [28]. Fortunately, it is shown in Reference [31] that improved ${\hat{B}}_{1}^{+}$ phase approximations can be obtained from the tranceive phase by incorporating an iterative-phase correction scheme in the CSI–EPT reconstruction algorithm. This correction scheme seems to reliably retrieve ${\hat{B}}_{1}^{+}$ phase maps from the measured tranceive phase, and leads to improved conductivity and permittivity reconstructions compared with reconstructions that are obtained when tranceive-phase approximation is applied. We will include this phase-correction mechanism in future CSI–EPT implementations as well. Another option is to opt for phaseless approaches as, for example, proposed in References [32,33].

Finally, in practice, the current densities in the transmit coil that generate the incident field depend on the present object, and we must account for this loading effect as well. Specifically, integral representations for the fields in CSI–EPT are obtained using scattered-field formalism in which it is assumed that current density in the transmitting antenna is impressed and independent of the scatterer that may be present in the configuration. In practice, however, these currents do depend on the object, and, consequently, care must be taken when we compute the background field in CSI–EPT. One approach is, therefore, to simulate this loading effect using a suitable coil and body model in a commercial field solver and to extract current densities in the coil from this solver. The background field in CSI–EPT (the field without any load) can then be computed using these extracted currents. In this way, the loading effect encountered in practice can be incorporated in our CSI–EPT reconstruction algorithm.

Our final aim is, of course, to turn CSI–EPT into a practical reconstruction method to obtain accurate and reliable conductivity and permittivity tissue maps of an interior part of the human body at MR operation frequencies. Reconstruction results based on simulated data are very promising, and we think that, by addressing the practical issues discussed above, we will indeed make significant progress towards a reliable EPT reconstruction method that provides us with accurate dielectric tissue maps in practice.

## Figures and Tables

**Table 1:** Mean and standard deviation of reconstructed electrical properties in different tissue that are apparent in the center slice of the head models using 2D CSI–EPT and foIC-EPT in a two-dimensional setting. Units of σ and $\varepsilon_{r}$ are in siemens per meter and permittivity of free space, respectively.

	Conductivity (σ)			Relative Permittivity ($\varepsilon_{r}$)		
	True	2D CSI–EPT	foIC-EPT	True	2D CSI–EPT	foIC-EPT
Fat	0.07	0.13 ± 0.10	0.18 ± 0.10	12.37	17.23 ± 7.89	19.27 ± 7.15
Red marrow	0.16	0.11 ± 0.06	0.15 ± 0.03	13.54	11.08 ± 2.52	13.68 ± 1.47
Bone	0.07	0.14 ± 0.15	0.21 ± 0.14	14.72	19.09 ± 8.05	21.73 ± 6.90
Eye lens	0.31	0.54 ± 0.11	0.78 ± 0.09	42.79	48.86 ± 5.26	51.52 ± 1.44
Nerve	0.35	0.74 ± 0.30	0.77 ± 0.26	44.07	51.49 ± 8.74	47.33 ± 7.42
Connective tissue	0.50	0.47 ± 0.12	0.44 ± 0.13	51.86	46.48 ± 7.56	40.54 ± 7.06
White matter	0.34	0.36 ± 0.04	0.38 ± 0.04	52.53	54.33 ± 3.12	55.37 ± 3.54
Muscle	0.72	0.63 ± 0.11	0.55 ± 0.13	63.49	56.76 ± 7.28	48.30 ± 8.72
Eye sclera	0.92	0.89 ± 0.15	0.87 ± 0.13	65.00	56.78 ± 6.67	50.26 ± 5.95
Skin	0.52	0.48 ± 0.08	0.36 ± 0.09	65.44	59.00 ± 8.95	42.29 ± 8.55
Hypothalamus	0.80	0.88 ± 0.11	0.91 ± 0.11	66.78	59.48 ± 4.91	54.12 ± 4.20
Eye vitreous humor	1.51	1.46 ± 0.13	1.40 ± 0.15	69.06	66.19 ± 4.86	61.03 ± 4.95
Cornea	1.06	0.92 ± 0.13	0.83 ± 0.11	71.46	61.52 ± 9.32	52.81 ± 5.84
Gray matter	0.59	0.61 ± 0.15	0.63 ± 0.15	73.52	72.68 ± 4.18	70.54 ± 4.92
Midbrain	0.83	0.84 ± 0.17	0.88 ± 0.18	79.74	78.58 ± 10.24	81.93 ± 12.33
Cerebrospinal fluid	2.14	1.90 ± 0.29	1.75 ± 0.29	84.04	80.66 ± 8.57	76.46 ± 11.31
Mucosa	2.28	1.50 ± 0.03	1.01 ± 0.02	116.00	78.07 ± 5.17	53.83 ± 2.77
